# Syndecan-1 antigen, a promising new target for triple-negative breast cancer immuno-PET and radioimmunotherapy. A preclinical study on MDA-MB-468 xenograft tumors

**DOI:** 10.1186/2191-219X-1-20

**Published:** 2011-09-01

**Authors:** Caroline Rousseau, Anne Lise Ruellan, Karine Bernardeau, Françoise Kraeber-Bodéré, Sebastien Gouard, Delphine Loussouarn, Catherine Saï-Maurel, Alain Faivre-Chauvet, John Wijdenes, Jacques Barbet, Joëlle Gaschet, Michel Chérel, François Davodeau

**Affiliations:** 1 Nuclear Medicine Dept, ICO René Gauducheau Cancer Center, IRCNA, Saint Herblain, France; 2 Centre de Recherche en Cancérologie de Nantes-Angers, Université de Nantes, Inserm, U892, Nantes, France; 3 Nuclear Medicine Dept, University Hospital, Nantes, France; 4 Pathology Dept, University Hospital, Nantes, France; 5 Gen Probe Diaclone, Besançon, France

**Keywords:** breast cancer, syndecan-1, CD138, radioimmunotherapy, immuno-PET, monoclonal antibody

## Abstract

**Background:**

Overexpression of syndecan-1 (CD138) in breast carcinoma correlates with a poor prognosis and an aggressive phenotype. The objective of this study was to evaluate the potential of targeting CD138 by immuno-PET imaging and radioimmunotherapy (RIT) using the antihuman syndecan-1 B-B4 mAb radiolabeled with either ^124^I or ^131^I in nude mice engrafted with the triple-negative MDA-MB-468 breast cancer cell line.

**Method:**

The immunoreactivity of ^125^I-B-B4 (80%) was determined, and the affinity of ^125^I-B-B4 and the expression of CD138 on MDA-MB-468 was measured *in vitro *by Scatchard analysis. CD138 expression on established tumors was confirmed by immunohistochemistry. A biodistribution study was performed in mice with subcutaneous MDA-MB-468 and ^125^I-B-B4 at 4, 24, 48, 72, and 96 h after injection and compared with an isotype-matched control. Tumor uptake of B-B4 was evaluated *in vivo *by immuno-PET imaging using the ^124^I-B-B4 mAb. The maximum tolerated dose (MTD) was determined from mice treated with ^131^I-B-B4 and the RIT efficacy evaluated.

**Results:**

^125^I-B-B4 affinity was in the nanomolar range (Kd = 4.39 ± 1.10 nM). CD138 expression on MDA-MB-468 cells was quite low (Bmax = 1.19 × 10^4^ ± 9.27 × 10^2^ epitopes/cell) but all expressed CD138 *in vivo *as determined by immunohistochemistry. The tumor uptake of ^125^I-B-B4 peaked at 14% injected dose (ID) per gram at 24 h and was higher than that of the isotype-matched control mAb (5% ID per gram at 24 h). Immuno-PET performed with ^124^I-B-B4 on tumor-bearing mice confirmed the specificity of B-B4 uptake and its retention within the tumor. The MTD was reached at 22.2 MBq. All mice treated with RIT (*n *= 8) as a single treatment at the MTD experienced a partial (*n *= 3) or complete (*n *= 5) response, with three of them remaining tumor-free 95 days after treatment.

**Conclusion:**

These results demonstrate that RIT with ^131^I-B-B4 could be considered for the treatment of metastatic triple-negative breast cancer that cannot benefit from hormone therapy or anti-Her2/neu immunotherapy. Immuno-PET for visualizing CD138-expressing tumors with ^124^I-B-B4 reinforces the interest of this mAb for diagnosis and quantitative imaging.

## Background

The expression of syndecan-1 in breast cancer is described as a poor prognostic factor. However, the potential of targeting this antigen for immuno-PET or radioimmunotherapy has not yet been investigated. Syndecans represent a four-member family of transmembrane cell-surface heparan sulfate proteoglycans. Their biological effects on adhesion, migration, and growth factor signaling are thought to be mediated by their binding to growth factors, including FGFs, VEGF, HGF, or ECM molecules, via their HS chains [1-3]. Syndecan-1, also named CD138, is expressed by normal epithelial cells but is also transiently expressed in condensing mesenchyma during embryonic morphogenesis [4]. The biological functions of syndecan-1 potentially affect several steps in tumor progression and facilitate metastasis [5]. A prognostic value has been assigned to changes in syndecan-1 expression in several cancer types, including breast, colorectal, gastric, pancreatic, prostate, lung, endometrial, and ovarian cancers, as well as squamous cell carcinoma of the head and neck and multiple myeloma [6,7]. Syndecan-1 appears particularly interesting for breast carcinoma radioimmunotherapy (RIT). The antigen is expressed by potentially aggressive breast carcinomas, and its expression is tightly associated with the absence of estrogen receptors (ER) as well as with a high Ki67 proliferation index [8]. Triple-negative (ER-negative, progesterone receptor (PR)-negative, HER2/neu not overexpressed) breast cancer (TNBC) represents approximately 15% of all breast carcinomas [9]. It generally occurs in women below the age of 50 years and is associated with a high risk of distant recurrence and death during the first 3 to 5 years of follow-up [10]. Cytotoxic chemotherapy is currently the only treatment available for TNBC patients, but most of them have chemoresistant rampant disease with a poor prognosis. However, novel targeted therapies have the potential to change its natural course [11]. Monoclonal antibody therapy is one such targeted therapy that may prove beneficial in the management of these breast cancer subtypes [9,12].

B-B4 is a murine IgG1 mAb targeting syndecan-1 that was originally developed for multiple myeloma by Wijdenes and colleagues [13]. The B-B4 mAb has been shown to have high specificity for syndecan-1, but it is not cytotoxic for myeloma cells [14]. Subsequently, one study reported that ^131^I-labeled B-B4 mAb induced cellular death in an *in vitro *multiple myeloma model [15].

Several radioimmunotherapy clinical trials have been performed for breast cancer treatment targeting Tag 72, mucin, CEA, and an adenocarcinoma antigen recognized by the ChL6 antibody [16-19]. However, objective tumor responses were obtained with limited toxicity compared to standard chemotherapy treatment of patients at the same disease stage. Repeated dosing with radiolabeled antibody or combination of RIT with other therapeutic agents might further improve RIT efficacy.

To date, there has been no study investigating the diagnostic and therapeutic potential of radiolabeled B-B4 mAb in breast carcinoma, particularly in TNBC cells such as the MDA-MB-468 cell line that shows many of the recurrent basal-like molecular abnormalities [20]. The aim of this study was thus to evaluate the biodistribution, toxicity, and RIT efficacy of the radioiodinated anti-CD138 antibody B-B4 in mice xenografted with the MDA-MB-468 TNBC cell line.

## Methods

### Cell line

The human TNBC cell line MDA-MB-468 was obtained from LGC Promochem (Molsheim, France). The MDA-MB-468 cell line was cultured in adherent-cell monolayers in RPMI medium (Gibco BRL, Cergy-Pontoise, France) supplemented with 10% bovine calf serum (Gibco BRL), 1% glutamine (L-glutamine 200 mM; Gibco BRL), and 1% antibiotic (penicillin 100 U/ml, streptomycin 100 U/ml; Gibco BRL). The human myeloma cell line U266 used for the radiolabeled B-B4 immunoreactivity assay was obtained from the American Type Culture collection (Rockville, MD, USA).

### Animal model

NMRI-nu (nu/nu) mice over 8 to 10 weeks of age were grafted subcutaneously in the right flank with 5 × 10^6^ MDA-MB-468 cells in 0.1 mL of PBS. The animals were housed in aseptic conditions. Lugol's 1% solution was added to drinking water (0.1 mL/L) 2 days before RIT and then 2 weeks after injecting the radioiodinated reagent. The mice were injected with radiolabeled mAb 24 days after MDA-MB-468 engraftment once the tumor volume had reached a mean volume of 108 ± 55 mm^3^. The mice were housed in our animal core facility according to ongoing national regulations issued by INSERM and the French Department of Agriculture. The experiments performed in this study were approved by the local veterinary services (license number B44.565).

### Antibodies and radiolabeling

The reference antibody was the B-B4 anti-CD138 mAb (IgG1). This mouse IgG1 was kindly provided by Diaclone (Besançon, France). The 7D4 mAb was used as the isotype-matched control in the biodistribution studies [21]. This mAb recognizes the CMH-peptide complex formed by peptide MAGE3 271-279 and HLA-A2. This epitope is not expressed on MDA-MB-468 cells. The antibodies were labeled with ^125^I (PerkinElmer 16, Avenue du Québec, Courtaboeuf Cedex 1, France) using the iodogen method [22]. The ^125^I-labeled mAb was purified on a PD10 column. The B-B4 mAb was also labeled with ^131^I (MDS Nordion, Zoning Industriel, Avenue de L'Espérance, Fleurus, Belgium) using the same iodogen method. The specific activity ranged from 185 to 200 MBq/mg. Radiolabeling efficiency estimated by instant thin-layer chromatography (ITLC) was above 95%. The ^131^I-labeled antibody was not purified further. Iodine-^124 ^(IBA molecular, Chemin du cyclotron, 3, Louvain-la-Neuve, Belgium)

labeling of the B-B4 mAb for immuno-PET imaging was performed according to a previously described method [22]. Briefly, 1.7 mg (350 μL) B-B4 in 0.1 M phosphate buffer pH 7 and 20 μl (0.1 mg) of iodogen in DMF were added to a glass vial containing 230 μL (222 MBq) of Na^124^I. After 15 min of incubation under gentle mixing at room temperature, the radiolabeled antibody was purified by gel filtration on NAPTM-5 columns (GE Healthcare UK, Little Chalfont Buckinghamshire, UK). The ^124^I-B-B4 was recovered in 1.15 ml. The specific activity was 90 mBq/mg, and the radiochemical purity of the purified radiolabeled antibody was greater than 98% as determined by ITLC-SG chromatography using trichloroacetic acid at 10% *w*/*v *in distilled water as a mobile phase.

### Immunoreactivity and affinity

The immunoreactivity of radiolabeled B-B4 was determined according to the "Lindmo" cell-binding assay using CD138-positive U266 cells [23]. The number of antigen-binding sites per MDA-MB-468 cells was determined by Scatchard analysis as previously described [21]. The binding data were subjected to nonlinear regression analysis using a one-site equilibrium binding equation with Prism software.

### Immunohistochemistry

Tumor sections (4 μm) were cut from the tissue microarray blocks and placed on superfrost slides. The immunochemical technique was performed with an automated immuno-stainer (Labvision, Fremont, CA, USA) using the strepatavidin-biotin amplification technique (ChemMate kit, Dako, Glostrup, Denmark) after appropriate antigen retrieval in EDTA buffer (pH 8) at 95°C. This involved the application of a specific primary antibody to syndecan-1/CD138 (clone MI15, 1:100, Dako). A secondary anti-mouse antibody conjugated to peroxidase was used for revelation. Peroxidase activity was revealed using 3,3'-diamino-benzidine for 5 min. Sections were counterstained with Harris hematoxylin for 3 min. Negative controls were obtained by omitting primary antibodies.

### Biodistribution

MDA-MB-468 tumor-bearing mice were given 4 MBq of ^125^I-labeled B-B4 (5 μg) via the tail vein. The mice were killed 4, 24, 48, 72, and 96 h after injection (three mice per time point), the tumor and organs were removed, weighed, and the radioactivity counted using a gamma counter. The results were expressed as percent ID per gram. The same protocol was applied for biodistribution of the control isotype-matched 7D4 mAb.

### PET imaging

Immuno-PET imaging with ^124^I-labeled B-B4 was performed on six NMRI-nu (nu/nu) mice bearing MDA-MB-468 xenografts. An activity of 3.3 MBq of ^124^I-labeled B-B4 (90 μg) was injected intravenously, and PET images were acquired 1, 2, 3, 4, and 8 days after injection, with an Inveon PET scanner (Siemens Medical Solutions, Knoxville, TN, USA) under anesthesia (isoflurane-O2). A CT scan was performed using the docked CT module (Siemens Medical Solutions, Knoxville, TN, USA).

PET imaging with ^18^FDG was performed on three NMRI-nu (nu/nu) mice bearing MDA-MB-468 xenografts receiving an intravenous injection of 6.2 MBq of ^18^FDG. PET images were acquired 1 h after injection. PET data were collected over a period of 20 min (one bed position), and the 3D sinograms were reconstructed using a 3D ordered subset expectation maximization followed by a maximum a posteriori algorithm (3D OSEM-MAP).

### Dose escalation and toxicity of ^131^I-labeled B-B4

The mice were injected i.v. in the lateral tail vein with escalating activity of ^131^I-labeled B-B4 mAb. The amount of B-B4 mAb was adjusted to 120 μg by adding unlabeled B-B4 antibody and the injected doses were adjusted to a constant volume of 0.2 ml with sterile PBS. These groups received 0.0 MBq (*n *= 4), 11.1 MBq (*n *= 4), 14.8 MBq (*n *= 4), 18.5 MBq (*n *= 4), 22.2 MBq (*n *= 4), or 25.9 MBq (*n *= 4) of ^131^I-labeled B-B4 mAb. This assay was repeated under the same experimental conditions with 25.9 (*n *= 3) and 37 MBq (*n *= 3). The maximum tolerated dose (MTD) was determined using the data obtained in the two assays as the dose just below the one at which at least one mouse died or lost more than 10% of its weight before treatment. The mice were weighed weekly for 96 days, and blood samples were taken from the inner border of the eye at 0, 14, 28, 40, 55, and 90 days after ^131^I-labeled B-B4 mAb injection. Leukocyte and platelet numbers were determined using a cell counter (Melet-Schloesing Laboratories, Cergy-Pontoise France).

### Radioimmunotherapy

An additional RIT experiment was performed under the same conditions as those described for the dose escalation at the MTD (22.2 MBq) (n = 4) and at a lower dose of 14.8 MBq (n = 4) compared to the control group (n = 5). Tumor volumes were measured with a sliding caliper twice a week for 96 days measuring tumor length (*L*), width (*w*) and thickness (*t*). Tumor volume (*V*) was calculated according to the formula: *V *= *π*/6 × *L *× *w *× *t*. In order to increase statistical power, the results of tumor growth assessed by this assay were pooled with those of the corresponding mice groups receiving the same doses in the dose escalation assay. The effect of unlabeled B-B4 mAb (120 μg) and the ^131^I-labeled isotype-matched mAb control was tested in comparison with a PBS control group in an independent assay.

The parameters used to evaluate the efficacy of each type of treatment were the minimal relative volume (the ratio of the smallest measured tumor volume to the initial tumor volume before treatment) and the event defined as the growth delay (the time required for the tumor to double in size after measurement on the day of treatment). The mean tumor volume was calculated for each group on each day of measurement. Tumor responses were categorized as follows: cure (tumor disappeared with no recurrence at the end of the study 96 days later), complete response (CR) (tumor disappeared for at least 7 days but later re-grew), partial response (PR) (tumor volume decreased by 50% or more for at least 7 days but then re-grew).

### Statistical analysis

Correlations between dose and degree of toxicity (body weight loss, platelets, leukocytes) or efficacy (tumor growth) were made using the nonparametric Spearman's test. Groups of interest (radioimmunotherapy doses) were also compared using the nonparametric ANOVA with Bonferroni correction. Curves for event-free survival (the time required for tumors to reach at least twice the initial volume) were calculated according to the Kaplan-Meier method and compared using the Log-rank test. All analyses were two-sided. *P *values < 0.05 were considered significant. Analyses were performed using SAS 9.1 (SAS Institute, Cary, NC, USA) and Stata 10.0 SE (StataCorp, College Station, TX, USA). The comparison of the mean tumor volume curves was performed using a nonparametric test with an on-line software from UCL Institut de Statistique (Louvain, Belgium) http://www.stat.ucl.ac.be/ISpersonnel/lecoutre/Tgca/french/help/help.htm.

## Results

### Immunoreactivity and affinity of radiolabeled B-B4 mAb

**Figure 1:**
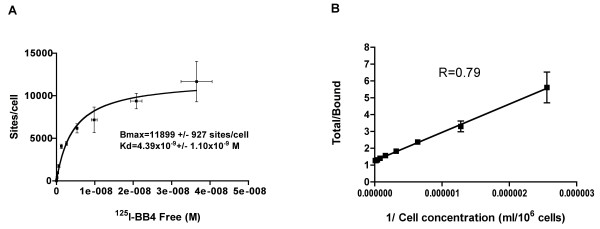
**Characterization of the B-B4 mAb**. **(A) **The affinity of B-B4 mAb and the number of antigenic sites on MDA-MB-468 breast cancer cells were evaluated by an equilibrium-binding assay with ^125^I-labeled B-B4 mAb. The nonspecific binding of ^125^I-labeled B-B4 mAb was evaluated with a tenfold excess of unlabeled B-B4 mAb at three concentrations of ^125^I-labeled B-B4 mAb and subtracted from binding data. The mean results of a triplicate assay are represented. The Bmax corresponding to the number of antigenic sites on MDA-MB-468 cells, and the Kd of ^125^I-labeled B-B4 were determined by nonlinear regression using a one-binding site equation. **(B) **The immunoreactivity of ^125^I-labeled B-B4 mAb was estimated by measuring the binding of a constant concentration of ^125^I-labeled B-B4 mAb to increasing numbers of U266 multiple myeloma cells that strongly expressed the CD138 antigen. This is a double-inverse plot of a triplicate assay. The fraction of immunoreactive ^125^I-labeled B-B4 (R) was determined as the inverse of the ordinate at the origin of the abscissa according to the Lindmo method [23].

CD138 expression was studied upon equilibrium binding of the specific B-B4 antibody to the human breast cancer MDA-MB-468 cell line. The expression of CD138 on this cell line was rather low; only 1.19 × 10^4 ^± 9.27 × 10^2 ^CD138 sites per cell were detected with saturating amounts of B-B4 mAb (Figure 1A). This result is consistent with previous work indicating that MDA-MB-468 is among the lowest syndecan-expressing breast cancer cell lines [24]. The affinity (dissociation constant) of B-B4 mAb was 4.39 ± 1.10 nM. The immunoreactivity assessed using the method described by Lindmo [23] (Figure 1B) was close to 80%, showing that the labeling did not modify the binding of B-B4 mAb to the CD138 antigen. For convenience, the immunoreactivity assay was performed on the multiple myeloma cell line U266 that expresses at least ten times more CD138 antigen than MDA-MB-468 cells.

### Immunohistochemistry

**Figure 2:**
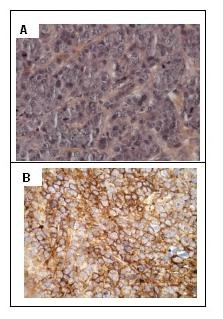
**Immunohistochemistry of a MDA-MB-468 breast tumor xenograft**. The expression of CD138 in MDA-MB-468 xenografts was assessed by immunohistochemistry. **(A) **Original magnification ×400 for hematoxylin-eosin staining. **(B) **Original magnification ×400 for CD138 immunostaining.

Syndecan-1 expression was further investigated by immunohistochemistry on xenograft tumors in order to validate the preclinical model for CD138 targeting. The lack of estrogen or progesterone receptor and Her2/neu expression on the MDA-MB-468 cell line was consistent with the capacity of this cell line to form tumors in nude mice without additional hormone treatment. Figure 2 presents micrographs of an MDA-MB-468 xenograft with hematoxylin-eosin staining (panel A) and CD138 immunostaining (panel B). The trabeculae of carcinoma cells were observed with high nuclear pleomorphism, high mitotic activity, and a central necrosis. Despite the relatively low expression of CD138 on cultured MDA-MB-468 cells, intense membrane immunostaining of carcinoma cells from tumor xenografts was visualized by CD138 immunostaining with the B-B4 mAb.

### Biodistribution of ^125^I-labeled B-B4 mAb

**Figure 3:**
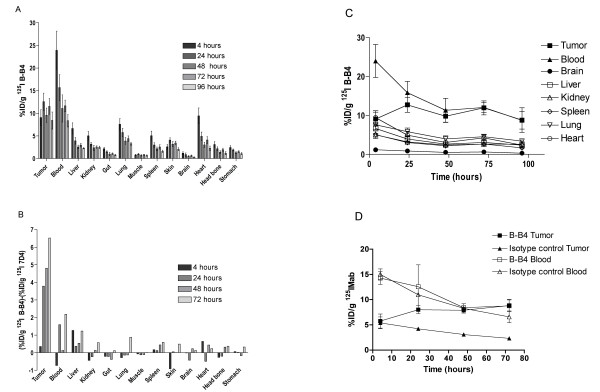
**Biodistribution of ^125^I-labeled B-B4 mAb**. **(A) **The biodistribution of ^125^I-labeled B-B4 mAb was estimated in nude mice engrafted with the MDA-MB-468 breast cancer cell line. The results are expressed as mean values ±95% confident intervals of the percentage of injected dose per gram (%ID/g) in the considered organ or tissue from three triplicate assays. **(B) **This panel shows the specific binding of the B-B4 antibody calculated by subtracting the %ID/g of the ^125^I-labeled 7D4 isotype-matched control antibody to that of ^125^I-labeled B-B4 measured in a triplicate assay under the same experimental conditions for both antibodies. (C) The %ID/g of ^125^I-labeled B-B4 mAb obtained from biodistribution studies at different time points are plotted versus time in order to visualize the kinetics of activity uptake in different tissues. (D) The kinetics of 7D4 and B-B4 distribution in the blood and tumors are shown in the same graph to demonstrate the identical blood pharmacokinetics of both antibodies and the specific uptake of BB4 in the tumor. The difference between the uptake of the two antibodies in the tumor is not significant at 4 h but is clearly significant at 24, 48, and 72 h (*p *< 0.001 determined by a two-way ANOVA test).

In mice injected with ^125^I-labeled B-B4 mAb, maximum tumor uptake was reached at 24 h (13.8% ± 1.8% ID per gram) and was maintained over time with a tumor uptake of 8.4% ± 1.9% ID per gram at 96 h (Figure 3A). In normal tissues, the maximum accumulation was observed at 4 h but rapidly decreased with kinetics similar to those for blood (Figure 3C). A higher level of ^125^I-labeled B-B4 mAb was observed in the more vascularized organs such as the heart and lungs, in keeping with their higher blood content. The level of ^125^I-labeled B-B4 mAb in the blood reflected the serum stability of radiolabeled B-B4 mAb.

We further investigated the specificity of B-B4 mAb tumor uptake by comparing it with the biodistribution of an isotype-matched control antibody. To this end, we used the 7D4 IgG1 antibody developed in our laboratory, which does not bind to MDA-MB-468 cells. Tumor uptake measured with the nonspecific 7D4 mAb was subtracted from that detected with the B-B4 mAb in the same assay. The results presented in Figure 3B clearly show that all organs except the tumor displayed an identical B-B4 and 7D4 mAb uptake, with differences that did not exceed 1% ID per gram. Conversely, a clear and specific binding of ^125^I-labeled B-B4 mAb was observed in the tumor. This specific ^125^I-labeled B-B4 mAb uptake increased rapidly on the first day post injection and at a lower rate up to 72 h thereafter (Figure 3D). The tumor uptake was long lasting since 7 days after injection, it reached at least 80% of the maximum measured at 72 h (data not shown). Nevertheless, B-B4 tumor uptake was relatively low as compared to other tumor-specific antibodies. Consequently, in this MDA-MB-468 tumor model, the tumor/blood ratio is almost 1:1, as compared to 2:1 in other models. These observations can be explained by the low CD138 expression by this breast cancer cell line, together with the central necrosis in the tumors at the time of the biodistribution study.

### Immuno-PET imaging

**Figure 4:**
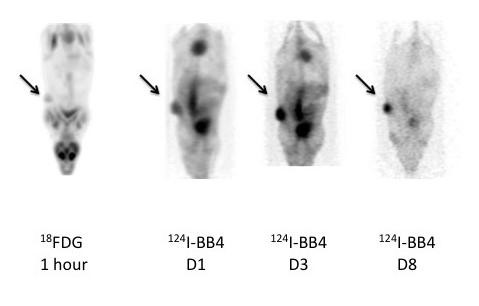
**Immuno-PET imaging with ^124I^-labeled B-B4 mAb. **PET image obtained 1 h after injection of 6 MBq ^18^F-FDG and at days 1, 3, and 8 after injection of 3.5 MBq of ^124^I-labeled B-B4 mAb.

The availability of isotopes of the same element, such as ^124^I and ^131^I or ^86^Y and ^90^Y, offers the possibility to image tumors by immuno-PET with the antibody used for therapy with identical labeling technology and, therefore, the same pharmacokinetics. This should afford more accuracy in dosimetry calculations before therapy and in providing information on tumor antigen expression complementary to metabolic ^18^FDG-PET imaging. We thus used the B-B4 mAb labeled with ^124^I for immuno-PET imaging in mice engrafted with MDA-MB-468 cells. Imaging was performed on days 1, 3, and 8 for mice injected with 3.5 MBq ^124^I-labeled B-B4 mAb or 1 h after ^18^FDG injection. Tumor imaging with ^124^I-labeled B-B4 was as informative as ^18^FDG for tumor visualization, even 1 day after antibody injection (Figure 4). Immuno-PET images recapitulated the results of biodistribution at all times, with a clear tumor imaging at day 1 together with an intense activity in the heart and blood vessels. At day 3, when antibody tumor uptake had reached its maximum, the staining was intense and comparable with that of the heart, which is in accordance with the equal uptake of the antibody in the blood and tumor observed at 72 h in the biodistribution study. Finally, at day 8, the persistence of the labeled antibody within the tumor, together with the decline in blood content, led to a well-contrasted image of the tumor with little signal remaining in normal tissues. This imaging assay validates the feasibility of syndecan-1 targeting for immuno-PET imaging with the B-B4 mAb.

### RIT toxicity and MTD

**Figure 5:**
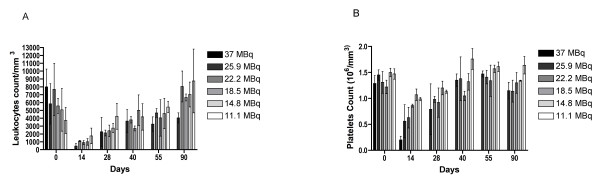
**Figure 5. Hematological toxicity of ^131^I-labeled B-B4 mAb. **Hematological toxicity was estimated by counting the leukocytes **(A)** and platelets **(B)** after treatment with increasing doses of ^131^I-labeled B-B4 mAb. All mice treated with 37 MBq died before day 14.

We performed a dose escalation study from 11.1 to 37 MBq with a 3.7-MBq increment of ^131^I-labeled B-B4 mAb. Hematological toxicity was assessed by platelet and leukocyte numeration (Figure 5). For platelets, the nadir was observed at day 14. A dose-dependent drop in platelet count was observed from 11.1 to 25.9 MBq reaching respectively 67.4% and 38.6% of the platelet number before treatment (0.56 × 10^6^/mm^3 ^vs. 1.45 × 10^6^/mm^3 ^and 0.99 × 10^6^/mm^3 ^vs. 1.47 × 10^6^/mm^3^, respectively). A more dramatic drop was observed at 37 MBq where the platelet count at day14 decreased to 15% of the pretreatment value, arguing for an acute hematological toxicity at this injected activity. The platelet count reverted to normal by day 40 at injected activities ranging from 11.1 to 25.9 MBq. The evolution of leukocyte count over time was similar to that of platelets, with a nadir at 14 days. The leukocyte count at injected activities ranging from 14.8 to 25.9 MBq decreased respectively to 19.7% and 7.9% of the pretreatment value (1,000 vs. 5,075/mm^3 ^and 460 vs. 5,847/mm^3^, respectively). An injected activity of 11.1 MBq was less toxic with a leukocyte count of 47.0% at day 14 and a rapid recovery of normal counts as early as 26 days after treatment. With the exception of the 37 MBq group in which all mice died, a progressive recovery in leukocyte number occurred after the nadir and a normal count was reached 70 days after injection of ^131^I-labeled B-B4 mAb. In summary, activities ranging from 11.1 to 25.9 MBq induced a dose-dependent hematological toxicity that spontaneously resolved in 40 days for platelets and 70 days for leukocytes. An injected activity of 37 MBq was clearly toxic.

The monitoring of animal survival and weight confirmed the mild toxicity of injected activities ranging from 11.1 to 22.2 MBq with no weight loss above 10% and no mortality during the follow-up period. Conversely, higher doses were toxic, with weight loss above 10% and death of all mice within 3 weeks of injection of 37 MBq ^131^I-labeled B-B4 mAb. In the 25.9-MBq group, one mouse out of seven died during the follow-up and two mice lost more than 10% of their initial weight. Therefore, we defined the 22.2-MBq injected activity of ^131^I-labeled B-B4 mAb as the MTD.

### RIT efficacy

**Figure 6:**
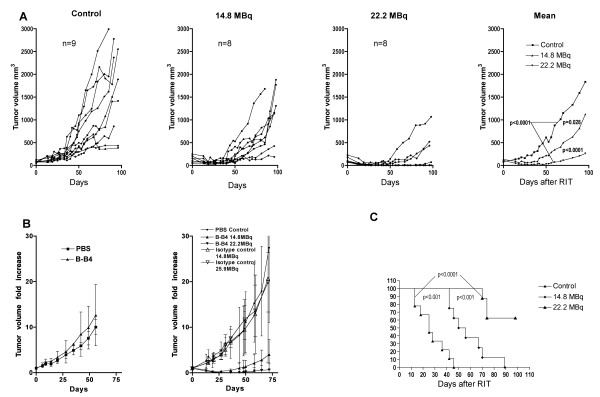
**Radioimmuntherapy assay**. **(A) **Tumor size variations in individual nude mice engrafted with the MDA-MB-468 cell line after treatment. Mice treated with 14.8 (n = 8) or 22.2 MBq (n = 8) of ^131^I-labeled B-B4 mAb can be compared with control mice (n = 9) that received unlabeled B-B4 mAb. The mean tumor volume of each group of mice is represented. The curves are very significantly different for the control versus 22.2 MBq (p < 0.0001) and the 14.8 MBq versus the 22.2 MBq groups (p < 0.0001) and significantly different for the control versus the 14.8 MBq groups (p = 0.028). **(B)** The effect of the unlabeled B-B4 mAb (n=5) and PBS (n=5) were tested in an independent experiment (left panel). The effect of RIT with 131I-labeled control isotype mAb (14.8, n=3 and 25.9 MBq, n=4) and 131I-B-B4 (14.8, n=4 and 22.2 MBq, n=4) and control PBS (n=4) were compared (right panel). Results are expressed as fold-increase in tumor volume compared to day 0. This figure summarizes the results of two independent assays performed under the same conditions. **(C) **The time required for tumors to double in size in groups given 14.8 and 22.2 MBq ^131^I-labeled B-B4 mAb compared with the control group..

The RIT assay was performed 24 days after engraftment of five million MDA-MB-468 cells when well-established tumors of a mean volume of 108 ± 55 mm^3 ^were obtained. The mice were treated with 22.2 or 14.8 MBq with a constant molar amount of B-B4. A clear tumor response was observed for the group of mice treated with 14.8 and 22.2 MBq of ^131^I-labeled B-B4 mAb, as compared to the control group (Figure 6A). Unlabeled B-B4 had no effect on tumor growth when used at the same molar dose as the ^131^I-labeled B-B4 mAb in the RIT assay. Equivalent activities of a nonspecific antibody were also tested but without a tumor response (Figure 6B). Despite individual variations in tumor growth within each group, the statistical analysis revealed a very significant difference in mean tumor volumes between control and 22.2-MBq-treated animals on the one hand and between 14.8- and 22.2-MBq-treated animals on the other hand (*p *< 0.0001). A significant difference between control and 14.8-MBq-treated animals (*p *= 0.028) was also observed, confirming the efficacy of ^131^I-labeled B-B4 mAb RIT (Figure 6A). In the group of eight mice that received 22.2 MBq ^131^I-labeled B-B4 mAb, five achieved a CR and three a PR (duration of 48 ± 32 days), and three mice were cured at the end of the assay. For the group of mice injected with 14.8 MBq of ^131^I-labeled B-B4 mAb, three out of eight treated mice achieved PR (duration of 31 ± 5 days), and all the mice had a tumor volume reduction within the 20 days following treatment, whereas tumor growth was continuous in the control group. We further analyzed the tumor response by comparing the growth delay (the time required for the tumor to double in size after measurement on the day of treatment) in the control, 14.8 MBq, and 22.2 MBq ^131^I-labeled B-B4 mAb-treated animals (Figure 6C). A statistically significant difference was found when groups were compared to each other, confirming the dose-dependent efficacy of ^131^I-labeled B-B4 mAb RIT treatment. The mean growth delays were 28 ± 13 days and 58 ± 17 days for the control group and the 14.8 MBq groups, respectively; whereas, in the group of eight mice treated with the 22.2 MBq dose, only three had a bigger tumor at the end of the follow-up than at the date of injection. Despite the low tumor/blood ratio, this assay demonstrated that targeting low CD138-expressing triple-negative breast cancer cells with ^131^I-labeled B-B4 mAb affords a very encouraging tumor response with only transient toxicity.

## Discussion

The MDA-MB-468 cancer cell line is representative of triple-negative breast cancer that has a poor prognosis. As such, it is often used as xenografts in nude mice to test new drugs and therapeutic strategies against breast cancer. Recent studies with a variety of drugs and combination therapies, including antimitotic agents and small molecules inhibiting several signaling pathways resulted in significant tumor growth control but in no real breakthrough. The same model was previously used for RIT studies of the RS7 antibody or its humanized equivalent hRS7 [25,26], with reported remission rates varying between 9% and 70%. The latter antibody recognizes a pancarcinoma antigen also known as EGP-1 or Trop2. In this paper, we show that when radiolabeled with appropriate iodine isotopes, the anti-CD138 (syndecan-1) antibody B-B4 shows favorable biodistribution, imaging capability, and antitumor properties in the same MDA-MB-468 xenograft model, with three PR, five CR, and three cured animals in a group of eight with established tumors, despite a relatively low expression of the target antigen. In fact, the equilibrium binding assay showed that the number of antigenic sites was as low as 1.2 × 10^4 ^per cell, which is consistent with previous reports [27,24]. This low level of CD138 expression explains the relatively low tumor/blood ratio found in the biodistribution analysis of the B-B4 antibody and the relatively high injected activity (22.2 MBq) required to cure mice from their tumor in the RIT assay. The treatment efficiency is probably explained by the long residence time of activity in the tumor. This was illustrated by immuno-PET imaging where the best tumor images were obtained 8 days after ^124^I-labeled B-B4 injection. No adverse effects were observed at the MTD defined as 22.2 MBq. Other groups targeted breast cancer xenografts using whole antibodies radiolabeled with ^131^I and have reported MTD ranging from 7.4 MBq for the ^131^I-labeled anti-Lewis Y hu3S193 antibody [28] to 44.4 MBq for the ^131^I-labeled anti-MUC-1 Mc5 antibody [29]. Interestingly, antibodies targeting the same antigen displayed a very different MTD. Behr and colleagues targeted ACE with the mouse IgG1 mAbs MN-14 and F023C5 and observed an equivalent RIT efficacy on GW-39 colon carcinoma, with an MTD of 9.62 and 22.2 MBq, respectively [30-32]. The 22.2 activity that we determined as the MTD for ^131^I-labeled B-B4 mAb is in the range of these previously described MTDs.

The choice of the radionuclide for imaging and therapy could influence the efficiency of these approaches. ^131^I presents some advantages, notably the possibility of imaging and dosimetry and a low activity uptake in the liver. ^131^I-labeled antibodies are successfully used in consolidation treatment of hepatic metastases of colon cancer after surgical removal of macroscopic lesions [33] or in the treatment of B cell lymphoma [34-36]. However, mAb labeled with iodine isotopes using the conventional chloramine-T method could result in a loss of efficacy due to the rapid escape of iodotyrosine resulting from the catabolism of the radioiodinated antibody within the lysosomal compartment, especially in the case of internalizing mAb. Several groups have reported that CD138 is internalized. However, the internalization mechanism has been described as clathrin-independent. Internalization has been shown to be fast and to involve a multistep process: ligand binding, clustering, energy-independent lateral movement into detergent-insoluble membrane rafts, and recruitment of actin and tyrosine kinases [37,38]. This mechanism has been studied by several groups using iodine-labeled ligands of CD138 or of a CD138-FcR fusion protein. Fast degradation of the ligands, like that usually observed for proteins endocytosed via clathrin-coated pits, does not seem to occur after CD138 internalization. In addition, our biodistribution data showed little decrease in tumor activity accumulation up to 96 h, while the circulating antibody concentration decreased at least threefold. In the present study of xenograft MDA MB, 468 PET imaging with ^124^I-labeled B-B4 confirmed the accessibility of the CD138 antigen and showed a good stability of tumor activity up to 8 days post injection. Thus, deiodination of the radiolabeled antibody seemed to be limited and does not preclude the possibility of performing radioimmunotherapy with ^131^I-B-B4. Although further studies are needed using a residualizing agent such as that described by Goldenberg and co-workers [26] or of radioactive metals such as lutetium-177 or yttrium-90 to increase the efficiency of RIT, here we show the potential of targeting CD138 for the treatment of breast cancer in the mouse model with a significant response rate despite the low number of antigen copies expressed by the triple-negative MDA-MB-468 cells.

Targeting CD138 for RIT is attractive since this antigen is associated with an aggressive breast cancer phenotype. CD138 is expressed by about 75% of ER-negative forms of breast cancer [39], and it is associated with a high histological grade, Ki 67 index, tumor size, and lymph node involvement [8,40]. RIT targeting CD138 could thus be very useful in the treatment of triple-negative breast cancer that is not eligible for hormone therapy or immunotherapy targeting Her2/neu. TNBC accounts for 10% to 17% of all breast carcinomas and among two thirds of TNBC patients express CD138 on their primary tumor. TNBC are chemosensitive, but TNBC patients with residual disease postchemotherapy have a poor outcome with an increased likelihood of distant recurrence [41]. Targeting tumor cells by RIT with ^131^I-labeled B-B4 mAb could offer the possibility to achieve responses on chemoresistant cancer cells for patients who relapse after intensive chemotherapy because ionizing radiations act differently on cancer cells. When considering breast cancer irrespective of the ER or PgR expression status, CD138 expression is associated with the worst prognostic marker Her2/neu. Barbareschi and colleagues reported on an additive adverse effect when both Her2/neu and CD138 were overexpressed [40]. Patients suffering from this kind of cancer can be treated by immunotherapy with herceptin associated to radioimmunotherapy with ^131^I-labeled B-B4 mAb in order to obtain a synergy between the two therapeutic approaches when used in association or as an additional line of treatment for patients who relapse.

It is generally accepted that RIT is more adapted to the systemic treatment of small tumors like metastasis. The expression of CD138 on primary breast tumors and in the corresponding invaded lymph node has been evaluated. The level of expression in invaded lymph nodes is at least equal to that of the primary tumor [24]. The shift of CD138 expression from epithelial cancer cells to fibroblasts of the stroma should not preclude RIT efficacy as long as the overall level of CD138 expression in the tumor ensures sufficient uptake. Indeed, the millimeter range of beta particles enables the irradiation of cells surrounding tumor epithelial cells via the cross-fire effect [42,43].

## Conclusion

RIT targeting CD138 is relevant for the treatment of triple-negative breast cancer and could be applied to patients who relapse after a first-line treatment. The possibility of visualizing tumors by immuno-PET and to perform quantitative imaging prior to therapy provides the advantage of being able to assess CD138 expression in order to conclude on the feasibility of RIT [18] for each patient.

## List of abbreviations

CD138: syndecan-1; ECM: extracellular matrix; ER: estrogen receptor; FGFs: fibroblast growth factors; HGF: hepatocyte growth factor; HS: heparan sulfate; PR: progesterone receptor; RIT: radioimmunotherapy; TNBC: triple-negative breast cancer; VEGF: vascular endothelial growth factor.

## Competing interests

The authors declare that they have no competing interests.

## Authors' contributions

CR carried out *in vivo *RIT experiments and immuno-PET. ALR carried out *in vivo *RIT and radiolabeled antibody quality control. KB carried out biodistribution studies. FK-B participated in the design of the study. SG carried out cell culture and participated in the *in vivo *assays. DL carried out immunohistochemistry on MDA-MB-468 tumors. CSM was responsible for the animal facility, participated in *in vivo *assays and in the radiolabeling of mAb. AF-C was responsible for high activity mAb radiolabeling. JW produced the B-B4 antibody and critically revised the manuscript. JB critically revised the manuscript and participated in drafting it. JG participated in the study design and was involved in drafting the manuscript. MC participated in the study design, critically revised the manuscript, and participated in drafting it. FD, corresponding author, designed and supervised the work.

## References

[B1] Beauvais DM Burbach BJ Rapraeger AC The syndecan-1 ectodomain regulates alphavbeta3 integrin activity in human mammary carcinoma cells J Cell Biol 2004 167 1 171 181 10.1083/jcb.200404171 15479743 PMC2172512

[B2] Bernfield M Gotte M Park PW Reizes O Fitzgerald ML Lincecum J Zako M Functions of cell surface heparan sulfate proteoglycans Annu Rev Biochem 1999 68 729 777 10.1146/annurev.biochem.68.1.729 10872465

[B3] Jakobsson L Kreuger J Holmborn K Lundin L Eriksson I Kjellen L Claesson-Welsh L Heparan sulfate in trans potentiates VEGFR-mediated angiogenesis Dev Cell 2006 10 5 625 634 10.1016/j.devcel.2006.03.009 16678777

[B4] Solursh M Reiter RS Jensen KL Kato M Bernfield M Transient expression of a cell surface heparan sulfate proteoglycan (syndecan) during limb development Dev Biol 1990 140 1 83 92 10.1016/0012-1606(90)90055-N 2358126

[B5] Ilan N Elkin M Vlodavsky I Regulation, function and clinical significance of heparanase in cancer metastasis and angiogenesis Int J Biochem Cell Biol 2006 38 12 2018 2039 10.1016/j.biocel.2006.06.004 16901744

[B6] Derksen PW Keehnen RM Evers LM van Oers MH Spaargaren M Pals ST Cell surface proteoglycan syndecan-1 mediates hepatocyte growth factor binding and promotes Met signaling in multiple myeloma Blood 2002 99 4 1405 1410 10.1182/blood.V99.4.1405 11830493

[B7] Yip GW Smollich M Gotte M Therapeutic value of glycosaminoglycans in cancer Mol Cancer Ther 2006 5 9 2139 2148 10.1158/1535-7163.MCT-06-0082 16985046

[B8] Baba F Swartz K van Buren R Eickhoff J Zhang Y Wolberg W Friedl A Syndecan-1 and syndecan-4 are overexpressed in an estrogen receptor-negative, highly proliferative breast carcinoma subtype Breast Cancer Res Treat 2006 98 1 91 8 10.1007/s10549-005-9135-2 16636895

[B9] Rakha EA El-Sayed ME Green AR Lee AH Robertson JF Ellis IO Prognostic markers in triple-negative breast cancer Cancer 2007 109 1 25 32 10.1002/cncr.22381 17146782

[B10] Haffty BG Yang Q Reiss M Kearney T Higgins SA Weidhaas J Harris L Hait W Toppmeyer D Locoregional relapse and distant metastasis in conservatively managed triple negative early-stage breast cancer J Clin Oncol 2006 24 36 5652 5657 10.1200/JCO.2006.06.5664 17116942

[B11] Oakman C Viale G Di Leo A Management of triple negative breast cancer Breast 2010 19 5 312 21 10.1016/j.breast.2010.03.026 20382530

[B12] Irvin WJ Jr Carey LA What is triple-negative breast cancer? Eur J Cancer 2008 44 18 2799 2805 10.1016/j.ejca.2008.09.034 19008097

[B13] Wijdenes J Vooijs WC Clement C Post J Morard F Vita N Laurent P Sun RX Klein B Dore JM A plasmocyte selective monoclonal antibody (B-B4) recognizes syndecan-1 Br J Haematol 1996 94 2 318 323 10.1046/j.1365-2141.1996.d01-1811.x 8759892

[B14] Couturier O Faivre-Chauvet A Filippovich IV Thedrez P Sai-Maurel C Bardies M Mishra Ak Gauvrit M Blain G Apostolidis C Molinet R Abbe JC Bataille R Wijdenes J Chatal JF Chérel M Validation of 213Bi-alpha radioimmunotherapy for multiple myeloma Clin Cancer Res 1999 5 10 Suppl 3165s 3170s 10541359

[B15] Supiot S Faivre-Chauvet A Couturier O Heymann MF Robillard N Kraeber-Bodere F Morandaeu L Mahé MA Chérel M Comparison of the biologic effects of MA5 and B-B4 monoclonal antibody labeled with iodine-131 and bismuth-213 on multiple myeloma Cancer 2002 94 4 Suppl 1202 1209 11877746 10.1002/cncr.10286

[B16] DeNardo SJ O'Grady LF Richman CM DeNardo GL Overview of radioimmunotherapy in advanced breast cancer using I-131 chimeric L6 Adv Exp Med Biol 1994 353 203 211 7527179 10.1007/978-1-4615-2443-4_19

[B17] Schrier DM Stemmer SM Johnson T Kasliwal R Lear J Matthes S Taffs S Dufton C Glenn SD Butchko G Ceriani RL Rovira D Bunn P Shpall EJ Bearman SI Purdy M Cagnoni P Jones RB High-dose 90Y Mx-diethylenetriaminepentaacetic acid (DTPA)-BrE-3 and autologous hematopoietic stem cell support (AHSCS) for the treatment of advanced breast cancer: a phase I trial Cancer Res 1995 55 23 Suppl 5921s 5924s 7493371

[B18] Behr TM Sharkey RM Juweid ME Dunn RM Vagg RC Ying Z Zhang CH Swayne LC Vardi Y Siegel JA Goldenberg DM Phase I/II clinical radioimmunotherapy with an iodine-131-labeled anti-carcinoembryonic antigen murine monoclonal antibody IgG J Nucl Med 1997 38 6 858 870 9189130

[B19] Mulligan T Carrasquillo JA Chung Y Milenic DE Schlom J Feuerstein I Paik C Perentesis P Reynolds J Curt G Goeckeler W Fordyce W Cheng R Riseberg D Cowan K O'Shauffnessy J Phase I study of intravenous Lu-labeled CC49 murine monoclonal antibody in patients with advanced adenocarcinoma Clin Cancer Res 1995 1 12 1447 1454 9815943

[B20] Oliveras-Ferraros C Vazquez-Martin A Lopez-Bonet E Martin-Castillo B Del Barco S Brunet J Menendez JA Growth and molecular interactions of the anti-EGFR antibody cetuximab and the DNA cross-linking agent cisplatin in gefitinib-resistant MDA-MB-468 cells: new prospects in the treatment of triple-negative/basal-like breast cancer Int J Oncol 2008 33 6 1165 1176 19020749

[B21] Bernardeau K Gouard S David G Ruellan AL Devys A Barbet J Bonneville M Cherel M Davodeau F Assessment of CD8 involvement in T cell clone avidity by direct measurement of HLA-A2/Mage3 complex density using a high-affinity TCR like monoclonal antibody Eur J Immunol 2005 35 10 2864 2875 10.1002/eji.200526307 16163672

[B22] Fraker PJ Speck JC Jr Protein and cell membrane iodinations with a sparingly soluble chloroamide, 1,3,4,6-tetrachloro-3a,6a-diphrenylglycoluril Biochem Biophys Res Commun 1978 80 4 849 857 10.1016/0006-291X(78)91322-0 637870

[B23] Lindmo T Boven E Cuttitta F Fedorko J Bunn PA Jr Determination of the immunoreactive fraction of radiolabeled monoclonal antibodies by linear extrapolation to binding at infinite antigen excess J Immunol Methods 1984 72 1 77 89 10.1016/0022-1759(84)90435-6 6086763

[B24] Burbach BJ Friedl A Mundhenke C Rapraeger AC Syndecan-1 accumulates in lysosomes of poorly differentiated breast carcinoma cells Matrix Biol 2003 22 2 163 77 10.1016/S0945-053X(03)00009-X 12782143

[B25] Shih LB Xuan H Aninipot R Stein R Goldenberg DM In vitro and in vivo reactivity of an internalizing antibody, RS7, with human breast cancer Cancer Res 1995 55 23 Suppl 5857s 5863s 7493360

[B26] Govindan SV Stein R Qu Z Chen S Andrews P Ma H Hansen HJ Griffiths GL Horak ID Goldenberg DM Preclinical therapy of breast cancer with a radioiodinated humanized anti-EGP-1 monoclonal antibody: advantage of a residualizing iodine radiolabel Breast Cancer Res Treat 2004 84 2 173 182 10.1023/B:BREA.0000018417.02580.ef 14999147

[B27] Gotte M Kersting C Radke I Kiesel L Wulfing P An expression signature of syndecan-1 (CD138), E-cadherin and c-met is associated with factors of angiogenesis and lymphangiogenesis in ductal breast carcinoma in situ Breast Cancer Res 2007 9 1 R8 10.1186/bcr1641 17244359 PMC1851383

[B28] Clarke K Lee FT Brechbiel MW Smyth FE Old LJ Scott AM Therapeutic efficacy of anti-Lewis(y) humanized 3S193 radioimmunotherapy in a breast cancer model: enhanced activity when combined with taxol chemotherapy Clin Cancer Res 2000 6 9 3621 3628 10999754

[B29] Peterson JA Blank EW Ceriani RL Effect of multiple, repeated doses of radioimmunotherapy on target antigen expression (breast MUC-1 mucin) in breast carcinomas Cancer Res 1997 57 6 1103 1108 9067279

[B30] Behr TM Memtsoudis S Sharkey RM Blumenthal RD Dunn RM Gratz S Wieland E Nebendahl K Schmidberger H Goldenberg DM Becker W Experimental studies on the role of antibody fragments in cancer radio-immunotherapy: influence of radiation dose and dose rate on toxicity and anti-tumor efficacy Int J Cancer 1998 77 5 787 795 10.1002/(SICI)1097-0215(19980831)77:5<787::AID-IJC19>3.0.CO;2-Z 9688314

[B31] Behr TM Wulst E Radetzky S Blumenthal RD Dunn RM Gratz S Rave-Fränk M Schmidberger H Raue F Becker W Improved treatment of medullary thyroid cancer in a nude mouse model by combined radioimmunochemotherapy: doxorubicin potentiates the therapeutic efficacy of radiolabeled antibodies in a radioresistant tumor type Cancer Res 1997 57 23 5309 5319 9393755

[B32] Behr TM Sgouros G Vougiokas V Memtsoudis S Gratz S Schmidberger H Blumenthal RD Goldenberg DM Becker W Therapeutic efficacy and dose-limiting toxicity of Auger-electron vs. beta emitters in radioimmunotherapy with internalizing antibodies: evaluation of 125I- vs. 131I-labeled CO17-1A in a human colorectal cancer model Int J Cancer 1998 76 5 738 48 10.1002/(SICI)1097-0215(19980529)76:5<738::AID-IJC20>3.0.CO;2-Z 9610734

[B33] Liersch T Meller J Bittrich M Kulle B Becker H Goldenberg DM Update of carcinoembryonic antigen radioimmunotherapy with (131)I-labetuzumab after salvage resection of colorectal liver metastases: comparison of outcome to a contemporaneous control group Ann Surg Oncol 2007 14 9 2577 2590 10.1245/s10434-006-9328-x 17570017

[B34] Leahy MF Turner JH Radioimmunotherapy of relapsed indolent non-Hodgkin lymphoma with 131I-rituximab in routine clinical practice: 10-year single-institution experience of 142 consecutive patients Blood 2011 117 1 45 52 10.1182/blood-2010-02-269753 20864582

[B35] Leahy MF Seymour JF Hicks RJ Turner JH Multicenter phase II clinical study of iodine-131-rituximab radioimmunotherapy in relapsed or refractory indolent non-Hodgkin's lymphoma J Clin Oncol 2006 24 27 4418 4425 10.1200/JCO.2005.05.3470 16940276

[B36] Jacene HA Filice R Kasecamp W Wahl RL Comparison of 90Y-ibritumomab tiuxetan and 131I-tositumomab in clinical practice J Nucl Med 2007 48 11 1767 1776 10.2967/jnumed.107.043489 17942813

[B37] Wilsie LC Gonzales AM Orlando RA Syndecan-1 mediates internalization of apoE-VLDL through a low density lipoprotein receptor-related protein (LRP)-independent, non-clathrin-mediated pathway Lipids Health Dis 2006 5 23 10.1186/1476-511X-5-23 16945147 PMC1592478

[B38] Fuki IV Meyer ME Williams KJ Transmembrane and cytoplasmic domains of syndecan mediate a multi-step endocytic pathway involving detergent-insoluble membrane rafts Biochem J 2000 351 Pt 3 607 12 11042114 PMC1221399

[B39] Leivonen M Lundin J Nordling S von Boguslawski K Haglund C Prognostic value of syndecan-1 expression in breast cancer Oncology 2004 67 1 11 18 10.1159/000080280 15459490

[B40] Barbareschi M Maisonneuve P Aldovini D Cangi MG Pecciarini L Angelo Mauri F Veronese S Caffo O Lucenti A Palma PD Galligioni E Doglioni C High syndecan-1 expression in breast carcinoma is related to an aggressive phenotype and to poorer prognosis Cancer 2003 98 3 474 83 10.1002/cncr.11515 12879463

[B41] Gopal AK Press OW Wilbur SM Maloney DG Pagel JM Rituximab blocks binding of radiolabeled anti-CD20 antibodies (Ab) but not radiolabeled anti-CD45 Ab Blood 2008 112 3 830 835 10.1182/blood-2008-01-132142 18502830 PMC2481554

[B42] Maeda T Desouky J Friedl A Syndecan-1 expression by stromal fibroblasts promotes breast carcinoma growth in vivo and stimulates tumor angiogenesis Oncogene 2006 25 9 1408 1412 10.1038/sj.onc.1209168 16247452

[B43] Mennerich D Vogel A Klaman I Dahl E Lichtner RB Rosenthal A Pohlenz HD Thierauch KH Sommer A Shift of syndecan-1 expression from epithelial to stromal cells during progression of solid tumours Eur J Cancer 2004 40 9 1373 1382 10.1016/j.ejca.2004.01.038 15177497

